# The use of hydroxyapatite toothpaste to prevent dental caries

**DOI:** 10.1007/s10266-021-00675-4

**Published:** 2021-11-22

**Authors:** Kelsey O’Hagan-Wong, Joachim Enax, Frederic Meyer, Bernhard Ganss

**Affiliations:** 1 Faculty of Dentistry, University of Toronto, 124 Edward St., Toronto, M5G 1G6 Canada; 2 Research Department, Oral Care Dr. Kurt Wolff GmbH & Co. KG, Johanneswerkstr, 34-36, 33611 Bielefeld, Germany

**Keywords:** Hydroxyapatite, Oral care, Enamel, Toothpaste

## Abstract

Dissolution of hydroxyapatite from the tooth structure at low pH can lead to the irreversible destruction of enamel and dentin, which if left untreated can result in pain and tooth loss. Hydroxyapatite toothpastes contain hydroxyapatite particles in micro- or nanocrystalline form that have been shown to deposit and restore demineralized enamel surfaces. As such, they are currently being explored as a fluoride-free anti-caries agent. This narrative review article aims to summarize the recent findings of the research investigating the remineralization potential of HAP toothpaste in vitro, in situ and in vivo, as well as some other applications in dentistry.

## Introduction

It is estimated that roughly 35% of the population has untreated dental decay [1]. While fluoride has been a cornerstone in the prevention of dental caries, there is need for effective alternatives due to the decreased acceptance among the public and the risk of fluorosis in children [2]. Hydroxyapatite (HAP; Ca_5_(PO_4_)_3_(OH)) containing toothpastes are fluoride-free alternatives which have been recently shown to be effective as anti-caries agents [3–5].

To understand the mechanism of action of HAP toothpaste, it is essential to understand the structure of enamel and how it breaks down during the caries process. The main inorganic component found in dental enamel is HAP which is a mineral composed of calcium and phosphate ions. HAP crystals are highly organized to form compact enamel rods which extend from the dentin enamel junction to the outer surface of tooth enamel which are surrounded by enamel interrod [6, 7]. This highly organized three-dimensional structure contributes to enamel’s ability to withstand force, resist microbial attacks, and reflect light [8].

Under normal conditions, there is a dynamic balance between HAP mineral in the tooth structure and in the oral fluids [9]. Demineralization refers to the net loss of calcium and phosphate ions from the tooth structure whereas remineralization is the addition of calcium and phosphate back into the enamel structure from supersaturated oral fluids (i.e., precipitation of calcium phosphate).

The caries process is multifactorial [10]. At the molecular level, it is characterized by the loss of mineral from the HAP lattice. Specifically, the metabolism of carbohydrates by oral bacteria causes an initial drop in pH as a result of lactic acid production [11]. When the pH of the oral environment drops below a critical value of 5.5 for enamel, the increased H^+^ ions couple with free calcium and phosphate ions to shift the equation to demineralization which cause the dissolution of hydroxyapatite crystal and resulting in the loss of calcium and phosphate from the tooth structure [8, 9]. Early carious lesions, also known as enamel white spot lesions, are non-cavitated and capable of remineralization. The white opaque appearance of the lesions is due to the increased porosity of demineralized enamel which in turn results in a change in the refractive index. White spot lesions which are visible on dried enamel are typically confined to the outer enamel and are classified as code 1 using the *International Caries Detection Assessment System* (ICDAS)[12]. White spot lesions which are visible without being dried are deeper into enamel and possibly to the level of dentinoenamel junction (DEJ) and ICDAS code 2 [12]. While these lesions are theoretically capable of remineralizing and arresting, if they are left untreated, they may progress into dentin (ICDAS code 3 and beyond); at which point definitive restorations are recommended.

One major limiting factor in remineralization has been identified as calcium and phosphate ion availability [6]. The predominant ingredient in HAP toothpaste is calcium and phosphate, in the form of HAP, which can be used in micro- or nanocrystalline form [5, 13]. At the nano and micro sized level, HAP particles resemble naturally occurring enamel apatite crystals [3, 14]. HAP particles have been shown to bind to the damaged enamel surface and fill in the porous surface irregularities in order restore the surface integrity [15]. In comparison to fluorides which are limited to only surface remineralization, HAP particles are able to penetrate into the deeper layers of the lesion [16, 17]. Naturally occurring enamel is comprised of 20–40 nm HAP nanoparticles and it has been suggested that the use of 20 nm HAP nanoparticles is effective at repairing damaged enamel [18]. The rationale for using HAP particles as a remineralization agent is grounded on the principal of dental biomimetics, which is the belief that dental materials should mimic natural properties of the tooth. A comparison of the effects of HAP and fluoride toothpaste is found in Table 1.

**Table 1:** Comparison of the effects of HAP and fluoride toothpastes

	Hydroxyapatite toothpaste	Fluoride toothpaste
Mechanism of Action	Deposition of HAP particles either in the micro or nanocrystalline form, which bind to enamel surfaces to fill in demineralized surface defects and micropores [14]	Improvement of the natural tooth surface remineralization process [65]
Remineralization of enamel/dentin	Reports of increased surface remineralization by SEM and light microscopy, surface hardness, and mineralization as measured by TMR of HAP toothpaste compared to fluoride toothpaste in vitro and in situ [66] Similar results were found in terms of surface hardness between HAP toothpaste and 1450 ppm fluoride toothpaste [3] The effects on dentin remineralization In situ studies demonstrate equivalency of HAP toothpaste to 500 ppm fluoride toothpaste as measured by TMR	Fluoride toothpaste can lead to a limited increase in remineralization at the outer tooth surface [19, 67]
Caries Prevention	Non-inferiority of 10% microcrystalline HAP toothpastes compared with fluoride toothpastes in caries progression as measured by ICDAS score progression in both primary and permanent dentition [4, 5]	The caries prevention of fluorides is well-established [68]
Source of calcium and phosphate	Does not rely on calcium and phosphate in saliva [6]	Relies on salivary flow for calcium and phosphate ions [67]
Adverse effects	No adverse health events have been reported [63]	Dental fluorosis, systemic toxicity leading to bone fracture [69], developmental neurotoxicity [70, 71] and gastric irritation when used in excess [72]
Whitening	HAP gel found to produce increased brightening effects in vitro and in vivo [53, 58, 59]	No significant difference in tooth shade score from baseline [73]
Sensitivity	Decreased dentinal hypersensitivity compared to fluoridated toothpaste and tubule occlusion [20, 56, 57]	No significant difference between fluoride toothpaste and placebo [62]

Synthetic HAP-containing toothpastes were first developed by the National Aeronautics and Space Administration (NASA) for astronauts who, in the absence of gravity, were losing mineral content in the bones and teeth[8]. In 1970, the Japanese company Sangi LTD purchased the rights to synthetic HAP from NASA and launched the first ever HAP toothpaste (Apadent, Sangi Co) which claimed that it could repair tooth enamel [15]. Since its approval as an anti-caries agent in 1993 in Japan, it has become available commercially in Europe in 2006 and in Canada in 2015 [8, 16, 19]. The effect of a variety of HAP-containing toothpaste formulations has since been tested on enamel remineralization experimentally under laboratory and in situ conditions as well as clinically.

The purpose of this narrative review was to determine the effectiveness of hydroxyapatite-containing toothpastes on enamel remineralization in vitro, in situ and in vivo*.*

### Study selection

A literature search was carried out using the PubMed and Scopus database. The following keywords were applied “Hydroxyapatite”, “Dentifrices”, “Caries”, “Remineralization”, “Toothpastes”. Relevant papers in English were included in the review based on the title and abstract. Additional articles were screened from the references of selected papers as appropriate.

### The effect of hydroxyapatite-containing toothpastes on enamel remineralization in vitro

At the microscopic level, the surface of enamel has been well documented [8, 13]. Using scanning electron microscopy (SEM), sound enamel appears to have a smooth and intact surface. Following acidic insults, demineralized enamel appears porous with irregularities or divots present at the surface [6]^.^ Brushing extracted teeth with HAP-containing toothpaste has been found to result in HAP crystals binding and restoring the tooth surface and essentially filling in the pitted areas, as visualized by SEM [6, 20–23] and light microscopy [24], while the use of fluoride containing toothpaste (1040 ppm F^−^), however, produced very little surface remineralization and failed to repair any of the enamel surface irregularities. Semi quantitative analysis of mineral content showing that samples treated HAP-containing toothpaste had significantly higher levels of calcium content compared to controls [25]. Surface hardness has been used as another proxy to evaluate mineral content in teeth [26]. When teeth become demineralized they are “soft” and are less able to withstand forces. A study by Poggio et al. examined the surface hardness of demineralized bovine incisors following the use of two HAP-containing toothpastes, one fluoridated toothpaste and one non-fluoridated toothpaste. The authors found HAP-containing toothpaste produced increases in enamel surface hardness that was comparable to that of regular fluoride (1450 ppm F^−^) containing toothpastes [26]. Similar in vitro findings were also observed by previous studies [27–33]. One study by Comar et al*.* employed a similar experimental method and examined cross-sectional in demineralized bovine enamel and root dentin specimens but reported no differences between hydroxyapatite toothpaste formulations and fluoride-free controls [34]. Under in vitro conditions (i.e., not in RCTs), ambivalent results favoring fluoride toothpaste over HAP toothpaste were also reported in terms of resistance to acid challenges [35], improving enamel surface roughness [36], and at remineralizing artificial enamel lesions [37][37]. Although, conflicting in vitro evidence exists, the studies mentioned above seem demonstrate that HAP-containing toothpaste is somewhat equivalent to fluoridated toothpastes at remineralizing enamel lesions. The effects on dentin remineralization, however, are unclear. In deeper lesions such as those extending into dentin, it is unknown whether HAP particles can diffuse to deeper lesion surfaces.

Tschoppe et al. [3] compared the remineralizing effects of different HAP toothpaste formulations using bovine incisors dentin and enamel lesions. Samples were brushed twice daily for five seconds for a total of two weeks using either the HAP or fluoride toothpaste and then analyzed using transverse microradiography (TMR). TMR is a high-resolution imaging technique that is capable of quantifying small changes in mineral content. The authors found that all three HAP toothpaste formulations produced a similar degree of remineralization in both enamel and dentin lesions which was significantly higher than that produced in the fluoridated toothpaste group [3]. The effect of HAP toothpaste on root dentin demineralization has also been investigated by [39], in which the authors compared HAP to fluoride toothpaste. This study showed decreased dentin demineralization of nano-HAP toothpaste in which the effect was improved in dose dependent fashion in the presence of fluoride. Although more studies are needed to assess the effect of HAP-containing products on dentin lesions, results from the study published by Tschoppe et al., and Amaechi et al., are promising.

### The effect of hydroxyapatite-containing toothpastes on enamel remineralization in situ and ex vivo

One of the limitations of in vitro study protocols is that brushing the teeth ex vivo with toothpastes for 1–3 min may result in a higher concentration of product due to the lack of dilutive effect that normally occurs with saliva [40]. These studies may therefore result in an exaggerated and unrealistic effect. To better study the effect of HAP-containing toothpaste under “real life” conditions, in situ experimental designs have been employed [41]. One such study was a double-blind randomized crossover trial by Amaechi et al. in which participants wore a fixed appliance containing a demineralized block and sound block of enamel positioned on the buccal surface of their lower molars [16]. Participants were instructed to brush morning and evening with either the 10% HAP-containing toothpaste or 500 ppm fluoridated toothpaste for four weeks. Under these experimental conditions, the enamel blocks are subjected to the patients’ natural oral environment consisting of saliva, biofilm and dietary carbohydrate. The outcomes of this study were lesion rate reduction and mineral content as measured by microradiography. The authors found no difference between HAP-containing toothpaste and fluoridated toothpaste at demineralizing enamel blocks, thus demonstrating the non-inferiority of HAP toothpaste under these study conditions [16]. A similar in situ experimental design employed by Najibfard et al. [42] and Souza et al. [43] demonstrated similar differences in mineral gain among the fluoridated toothpaste control group and the nano-hydroxyapatite toothpaste groups using TMR analysis.

In addition, a study by Lelli et al. [44] examined the effects of HAP-containing toothpaste compared to fluoride containing toothpaste under ex*–*in vivo conditions. Participants were randomized to use either a 5% KNO_3_/NaF toothpaste with 1450 ppm F^−^ or a zinc (Zn)-substituted carbonate-hydroxyapatite toothpaste for 8 weeks. In this study, five of the patients in the experimental group and five of the patients in the control group were scheduled to undergo extractions of sound teeth for orthodontic or prosthetic reasons, which allowed the investigators to examine the enamel surface by SEM. The teeth from subjects who used the HAP-containing toothpaste had HAP deposits present on the enamel surface whereas those from subjects who used the fluoridated toothpaste showed no mineral surface deposition. Moreover, the HAP-containing toothpaste deposited only in the areas of demineralization and appeared to “repair” them. Similar SEM and hardness findings were found in extracted deciduous teeth following the use of HAP-containing toothpaste three times daily for 15 days [45]. Findings of these in vitro and in situ studies are found in Table 1.

### The effect of hydroxyapatite-containing toothpastes on enamel remineralization in vivo

HAP-containing toothpaste has proven to have high efficacy, however, there are very few published clinical studies demonstrating its effectiveness. A review of literature identified three prospective studies investigating the effect of HAP toothpaste as an anti-caries agent on caries incidence.

One of the first studies published followed a cohort of 181 Japanese school children (grade 4) from 1983 to 1986 [46]. Three different schools were included in the study; one school received a 5% HAP-containing toothpaste and the two others received control toothpastes. School children were instructed to brush their teeth at lunchtime with the given toothpaste using their normal brushing technique. Intraoral exams were performed at baseline and annually using an explorer and mirror and decayed, missing, filled, teeth (DMFT) were recorded for each patient. The authors found that mean DMFT indices of total erupted teeth were significantly lower in the HAP-containing dentifrice group compared to those in non-HAP placebo toothpaste group. The caries inhibition rate was found to be 35.86% in boys and in 55.93% in girls. A similar study by Shimura et al., in school children, reported a caries inhibition rate on DMFS was 26.42% after 1 year of HAP toothpaste use, indicating a statistically significant difference compared to control groups [16, 47].

A more recent clinical trial performed at five different German University Hospitals by Schlagenhauf et al. [5] compared the effectiveness of twice daily use of HAP toothpaste to fluoridated (1400 ppm; 350 ppm of AmF/1050 ppm of SnF_2_) toothpaste at preventing caries in high caries risk population undergoing fixed orthodontic treatment. Participants were randomized to either the HAP or fluoride toothpaste group and buccal surfaces of teeth were evaluated according using ICDASII criteria at baseline, and after 28, 56, 84, 112, 140, 168 days. The plaque and gingiva indices were also recorded at each visit. At the end of the 168-day period, 54.7% of the HAP group patients and 60.9% of the fluoride control group patients developed at least one ICDAS lesion ≥ code 1. While the results at each time period demonstrated a trend favoring the HAP group, the differences between the two groups were not statistically significant. In any case, this trial demonstrates the non-inferiority of HAP toothpaste compared to the fluoridated toothpaste at preventing caries in high-risk patients undergoing fixed orthodontic treatment [5]. Similar findings favoring the use of HAP toothpaste over fluoridated at restoring the appearance of post orthodontic white spot lesions was found by Badie et al. [48]. A short clinical trial by Makeeva et al. [49] also reported that improved sensitivity, enamel remineralization rate and acid resistance was found after only 3 months of use with nanohydroxyapatite toothpaste.

Of the most recent clinical trial investigating the effects of a microcrystalline HAP toothpaste to fluoridated toothpaste on caries progression in school children was published by Paszynska et al. [4]. The results of this one-year randomized clinical trial suggest that HAP is as efficacious as fluoridated toothpaste in treating dental caries in young children. This study controlled for baseline differences in caries experience and followed the children at six time points for evidence of disease progression. After the 1-year follow-up period, 72.2% of children in hydroxyapatite toothpaste group demonstrated development or progression of initial caries compared to 74.2% of children in the fluoridated control group [4]. A second randomized clinical trial that was published this year included 130 children and investigated the effect of HAP-containing products on enamel white spot lesions in primary teeth as measured by laser fluorescence [50]. The authors of this study found that compared to baseline, the HAP toothpaste group produced a significant difference in units of fluorescence, suggesting a remineralization that was comparable to that of fluoride containing MI paste and superior to that of fluoridated toothpaste (1450 ppm fluoride) [50]

### Additional effects of hydroxyapatite-containing toothpastes

The effect of HAP formulations on the oral biofilm has also been recently reviewed [41, 51, 52]. In one of the reviewed studies, a HAP-containing rinse was comparable to chlorhexidine rinses in reducing bacterial adhesion to the enamel surface. These findings are resonated by in vitro studies which demonstrate the inhibitory effects of zinc carbonate HAP on biofilm formation by *Streptococcus mutans* [53]. Interestingly, in the context of these studies HAP formulations did not kill any bacteria present in the biofilm compared to other bactericidal agents like chlorhexidine [51]. The use of HAP formulations may therefore have the advantage of preserving the normally occurring oral flora and increasing available mineral content on the tooth surface while also preventing colonization of pathogenic bacteria [54]. HAP toothpastes have also shown promising results at impeding bacterial adherence to enamel and lowering early biofilm formation [55].

In addition to preventing decay, several studies are beginning to demonstrate the effect of HAP-containing toothpaste as both a desensitizing and whitening agent [53]. Randomized clinical trial demonstrated that participants reported significantly lower levels of dentinal hypersensitivity in response to air blast after two and four weeks of HAP-containing toothpaste use compared to fluoridated toothpaste control subjects [56] and has been found to occlude dentinal tubules at the microscopic level [20, 57]. Niwa et al. found that HAP-containing toothpastes produced significant whitening and brightening effects on enamel in dose dependent fashion [58]. Improvement of objective measures of color change was observed using spectrophotometry color following the use of HAP-containing whitening gel used for 9 days [59].

One study, which examined the effect of HAP toothpaste at restoring the esthetics of white spot lesions, found that only ICON resin infiltration produced a significant improvement in color over that of HAP toothpaste [60, 61]. Results of a meta-analysis suggest that nano-HAP toothpastes may be superior as a desensitizing agent for the treatment of dentin hypersensitivity compared to calcium sodium phosphosilicate-containing, potassium-containing, strontium-containing, and fluoride toothpastes [62] (Table 1).

As mentioned previously, one of the major concerns with using fluoridated toothpaste on children under the age of 6 years is the development of dental fluorosis. There has also been concern over systemic toxicity of fluorides. Thus far in the literature, there have been no reports of adverse dental or systemic effects of HAP-containing toothpastes because HAP has an excellent biocompatibility [63]. Accidentally ingested calcium phosphate particles are dissolved in the stomach into their calcium and phosphate ionic form and have no adverse effects under realistic doses. Eukaryotic cell culture studies have shown that ingested HAP can lead to elevated intracellular levels of calcium ions, but that the cells are quickly able to regulate and return to normal calcium levels [56]. Lastly, calcium phosphate is naturally occurring mineral on in teeth and bones and there are no reported adverse effects on the environment [63].

## Conclusion

In vitro and in situ studies are demonstrating promising results of HAP toothpastes on the remineralization of enamel lesions and preventing/reducing demineralization. Specifically, research appears to demonstrate either its superiority or equivalency to fluoride toothpaste as anti-caries agents. Although there are a small number of randomized clinical trials available on this topic, which remains the gold standard for testing the effectiveness of interventions, the results of current in vivo studies are promising [4, 5, 64].

In conclusion, HAP is a biomimetic oral care agent, and its caries prevention has been tested in vivo, in situ, and in vitro with a high safety profile and no risk of fluorosis. While more research is needed to confirm the clinical effectiveness of HAP at preventing and arresting dental caries, the research suggesting its equivalency to fluoride toothpaste is promising. HAP-containing oral products can be considered as an alternative in young children where fluorosis is a concern. In addition to perhaps reducing the need for traditional dental restorations, HAP also offers relief from dentin hypersensitivity and reduces biofilm formation making it a multifunctional agent for preventive oral health care.
